# Impact of Matrix Surface Area on Griseofulvin Release from Extrudates Prepared via Nanoextrusion

**DOI:** 10.3390/pharmaceutics13071036

**Published:** 2021-07-07

**Authors:** Meng Li, Casey Furey, Jeffrey Skros, Olivia Xu, Mahbubur Rahman, Mohammad Azad, Rajesh Dave, Ecevit Bilgili

**Affiliations:** 1 Otto H. York Department of Chemical and Materials Engineering, New Jersey Institute of Technology, Newark, NJ 07102, USA; ml262@njit.edu (M.L.); cgf4@njit.edu (C.F.); jas239@njit.edu (J.S.); mr485@njit.edu (M.R.); dave@njit.edu (R.D.); 2 Department of Organismic and Evolutionary Biology, Harvard College, Cambridge, MA 02138, USA; oliviaxu@college.harvard.edu; 3 Department of Chemical, Biological and Bioengineering, North Carolina A&T State University, Greensboro, NC 27411, USA; maazad@ncat.edu

**Keywords:** nanocomposites, amorphous solid dispersion, wet media milling, nanoextrusion, wettability, dissolution

## Abstract

We aimed to examine the impact of milling of extrudates prepared via nanoextrusion and the resulting matrix surface area of the particles on griseofulvin (GF, a model poorly soluble drug) release during in vitro dissolution. Wet-milled GF nanosuspensions containing a polymer (Sol: Soluplus^®^, Kol: Kolliphor^®^ P407, or HPC: Hydroxypropyl cellulose) and sodium dodecyl sulfate were mixed with additional polymer and dried in an extruder. The extrudates with 2% and 10% GF loading were milled–sieved into three size fractions. XRPD–SEM results show that nanoextrusion produced GF nanocomposites with Kol/HPC and an amorphous solid dispersion (ASD) with Sol. For 8.9 mg GF dose (non-supersaturating condition), the dissolution rate parameter was higher for extrudates with higher external specific surface area and those with 10% drug loading. It exhibited a monotonic increase with surface area of the ASD, whereas its increase tended to saturate above ~30 × 10^−3^ m^2^/cm^3^ for the nanocomposites. In general, the nanocomposites released GF faster than the ASD due to greater wettability and faster erosion imparted by Kol/HPC than by Sol. For 100 mg GF dose, the ASD outperformed the nanocomposites due to supersaturation and only 10% GF ASD with 190 × 10^−3^ m^2^/cm^3^ surface area achieved immediate release (80% release within 30 min). Hence, this study suggests that ASD extrudates entail fine milling yielding > ~200 × 10^−3^ m^2^/cm^3^ for rapid drug release, whereas only a coarse milling yielding ~30 × 10^−3^ m^2^/cm^3^ may enable nanocomposites to release low-dose drugs rapidly.

## 1. Introduction

The dissolution performance of poorly water-soluble drugs can be improved using a variety of techniques, such as cocrystallization [1,2], amorphous solid dispersions (ASDs) [3,4], solid solutions [5,6], salts [7,8], micronization [9,10], and nanonization, a.k.a. nanomilling [11,12]. Among these techniques, nanomilling increases the surface area via size reduction of the drug crystals down to nanoscale, and their incorporation/encapsulation in a polymeric matrix yields nanocomposites that exhibit faster drug release [13,14,15]. On the other hand, dispersion of the drug in a polymer in amorphous form (ASD) increases the kinetic solubility [13]. Despite offering several advantages, such as enhanced dissolution rate, improved bioavailability, elimination of food effects, and their common use in marketed products [16], drug nanocomposites offer limited bioavailability enhancement for drugs with very low aqueous solubility [17]. Contrarily, drug ASDs provide significant dissolution improvement for such drugs. The amorphous form of the drug improves the extent–rate of dissolution and bioavailability of poorly water-soluble drugs because it could provide supersaturation in vivo and in vitro in the dissolution medium [18,19]. Compared to its crystalline counterpart, an amorphous drug exhibits higher “kinetic solubility” owing to its high free energy and metastable nature [20]. Despite this beneficial aspect, the amorphous drug can undergo precipitation upon wetting–dissolution of ASD particles, which detrimentally affects its physical stability and sustained supersaturation capability [20,21]. Supersaturation is desirable for the absorption and bioavailability of the drug if it is sustained over a gastrointestinal transition time of 4–5 h. Supersaturation, albeit desirable on its own, is also the driving force for primary or secondary nucleation (in the presence of residual crystals) [22], followed by growth and aggregation of precipitated particles, which can cause depletion of initially high supersaturation or a lower extent of dissolution [20,21,23,24,25]. Depending on their concentration and interactions with the drug, polymers in ASDs can inhibit drug precipitation, affording the sustainment of the drug supersaturation [23,24] besides their role in ASD formation through polymer–drug miscibility. Similarly, polymers also play an important role in drug nanocomposites; they have been used both as stabilizers during drug nanoparticle formation and as matrix formers [11,14,15].

Drug nanoparticles can be produced via “bottom-up” or “top-down” technologies, or their combinations [26,27]. They are usually in the form of suspensions, i.e., drug nanoparticles suspended in stabilizer solutions. To meet high patient/clinical demand for solid dosage forms, drug nanosuspensions are usually dried to form nanocomposite microparticles, i.e., drug nanoparticles dispersed/embedded in large micron-sized polymeric matrices, which are referred to as the nanocomposites or crystalline solid dispersions. Nanocomposite powders are usually incorporated into standard solid dosage forms, such as capsules, tablets, and sachets [28,29,30]. Drying of nanosuspensions can be achieved via spray drying [31,32], spray-freeze drying [33,34], freeze drying [35,36], vacuum drying [37,38], granulation with, or coating onto, inert excipient particles [28,39] as well as nanoextrusion recently [40,41]. Unlike nanocomposites, drug ASD is a single-phase amorphous mixture in which the drug is molecularly dispersed within a polymer matrix due to good polymer–drug miscibility and molecular interactions [42]. Processes for the preparation of ASDs can be categorized into two general types: solvent methods, such as spray drying [43], and fusion methods, such as hot melt extrusion (HME) [44]. Nanoextrusion was developed as a continuous drying process to convert a wet-milled drug nanosuspension along with additional dry polymer into nanocomposites via extrusion [40,45]. The drug nanoparticles are dispersed in the polymeric matrix while water evaporates, which yields extrudates in the form of nanocomposites. Besides handling viscous drug nanosuspensions and preparing extrudates with good content uniformity [46,47], nanoextrusion can also produce drug ASDs [41].

Extrusion processes used for the pharmaceutical manufacturing of solid dosage forms, including the traditional HME process and the recently developed nanoextrusion process, involve milling of the extrudate threads into powders for further downstream processing [48]. Modulating the particle (matrix) size via milling of extrudates potentially allows for manipulating the drug dissolution performance because matrix size could affect drug release from the extrudates significantly [41,48]. In most investigations, where extrudates in the form of ASDs were produced via HME, the extrudates were dry milled and sieved into a single size fraction. For example, Fule et al. [44] prepared an ASD of artesunate in two different matrices, i.e., Soluplus^®^ and Kollidon^®^ VA64, and the extrudates were milled and passed through a 200-µm sieve for various characterizations. Similarly, Juluri et al. [49] produced ASDs of griseofulvin and caffeine anhydrous in the matrix of kleptose linecaps DE17, and the milled extrudates between #40 and #50 sieves (297–420 µm) were collected and characterized. Pudlas et al. [50] produced an ASD of ibuprofen in the matrices of Soluplus^®^ and copovidone, and the milled extrudates were sieved to exclude particles larger than 250 µm. Javeer and Amin [51] produced an ASD of carbamazepine in the matrix of hydroxypropyl methyl cellulose, and the extrudates were milled and passed through a #40 sieve with opening size of 420 µm. Moreover, Nagy et al. [52] produced an ASD of spironolactone in the matrix of Soluplus^®^ and the milled extrudate was passed through a 300 µm sieve. Other studies reported *D*_10_, *D*_50_, and *D*_90_ or mean particle size of the extrudate particles (e.g., [53,54,55]). In most of the published literature, the actual detailed size statistics of the extrudate particles (matrix) in the sieved fraction were not measured or reported. More importantly, the impact of matrix size in various sieved size fractions on drug release has not been extensively examined and related to the specific surface area of the respective size fractions. Two recent studies [56,57] regarding matrix size effect on drug release from ASDs prepared by HME and melt-quenching will be discussed in Section 3.4 of this paper. When nanocomposites were produced by nanoextrusion, the impact of matrix size has not been investigated [40,47] or has not been examined extensively and systematically and analyzed for multiple size fractions and drug loadings [41].

The aim of this study is to examine the impact of milling and resulting matrix surface area while also elucidating the roles of drug loading, drug wettability, and polymer–drug miscibility, which modulate the solid state of the drug, on in vitro drug release from extrudate powders prepared via nanoextrusion. Griseofulvin (GF), an antifungal drug (GF), was selected as a model Biopharmaceutics Classification System (BCS) Class II drug because it is a challenging drug: it crystallizes fast [24] and its nanoparticles are prone to severe aggregation if not properly stabilized [39]. Three amphiphilic polymers, i.e., Kolliphor P407 (Kol), Hydroxypropyl cellulose (HPC), and Soluplus^®^ (Sol), along with sodium dodecyl sulfate (SDS) were selected to stabilize wet-milled GF suspensions and form matrices of the extrudates. GF has different miscibility with these polymers; the polymers are also expected to bring different aqueous wettability enhancement to the drug. The wet-milled drug suspensions along with additional polymer (Sol/Kol/HPC) were fed to a co-rotating twin-screw extruder, which dried the suspensions and formed extrudates. The extrudates with various polymer formulations and two target drug loadings (2% and 10% *w*/*w*) were dry-milled and sieved into three size fractions to examine the impact of matrix surface area. The drug nanosuspensions were characterized via laser diffraction, while the extrudates and their milled powders were characterized by laser diffraction, thermogravimetric analysis (TGA), scanning electron microscopy (SEM), and X-ray powder diffraction (XRPD). Digital microscopy was used to visualize the changes of different polymeric matrices when exposed to water. Drug wettability enhancement by Sol, Kol, and HPC solutions was studied using the modified Washburn method. Solvent-shift method was used to characterize the precipitation inhibiting capability of the polymers. Two drug doses, i.e., 8.9 and 100 mg, were used to investigate the dissolution response under *non-supersaturating* (low GF dose) *and supersaturating conditions* (high GF dose) in the bulk dissolution medium, respectively. The analysis of the experimental findings is expected to generate new insights into the roles of matrix surface area in drug release from the milled extrudates, while informing future studies about the potentially different milling needs (surface area of the extrudate) for various ASD and nanocomposite applications.

## 2. Materials and Methods

### 2.1. Materials

British Pharmacopoeia/European Pharmacopoeia (BP/EP) grade griseofulvin (GF) was purchased from Letco Medical (Decatur, AL, USA). GF is a BCS Class II drug with an aqueous solubility of 14.5 mg/L at 37 °C, melting point of 220 °C, and a glass transition temperature of 89 °C [58]. Soluplus^®^ (Sol, BASF, Tarrytown, NY, USA), Kolliphor^®^ P407 (Kol, BASF, Tarrytown, NY, USA), and Hydroxypropyl cellulose (HPC, SL grade, Nisso America Inc., New York, NY, USA) were used as polymeric stabilizer during wet media milling and polymeric matrix former during nanoextrusion. HPC is an amorphous polymer with two softening points at 68 and 178 °C [59]. Kolliphor^®^ P407 is a crystalline nonionic triblock copolymer composed of a central hydrophobic chain of polyoxypropylene flanked by two hydrophilic chains of polyoxyethylene with a melting temperature at 56 °C [60]. Soluplus^®^ is an amphiphilic polyvinyl caprolactam–polyvinyl acetate–polyethylene glycol graft copolymer. It is amorphous with a single glass transition temperature of 73 ± 2 °C [61]. Sodium dodecyl sulfate (SDS, GFS Chemicals, Inc., Columbus, OH, USA) was used as an anionic surfactant to enhance drug wettability and stabilize drug particles during milling. Its critical micelle concentration in water is 0.23 wt.% at ambient temperature [62]. Methanol (ACS reagent, ≥ 99.8%), purchased from Fisher Scientific (Suwanee, GA, USA), was used as a solvent. Yttrium stabilized zirconia beads (Zirmil Y, Saint Gobain ZirPro, Mountainside, NJ, USA) with a median size of 430 µm were used as the media in wet milling experiments.

### 2.2. Methods

#### 2.2.1. Wet Stirred Media Milling Process

Table 1 presents the formulations of the GF suspensions. Selection of the milling conditions and formulations was guided by our prior work on wet media milling [63,64]. GF powder was dispersed in an aq. stabilizer solution to prepare a 397 g suspension with 22.4% GF, 1.9% polymer (Sol/Kol/HPC), and 0.15% SDS. All percentages (%) refer to *w/w* with respect to the suspension. A Microcer wet stirred media mill (Netzsch Fine Particle Technology, LLC, Exton, PA, USA) with 80 mL chamber was used to mill the drug suspensions. Feed suspensions were milled for 120 min under identical conditions to those reported in Bilgili and Afolabi [63]: bead loading of 196 g, suspension flow rate of 126 mL/min, and stirrer (rotor) speed of 3200 rpm corresponding to a tip speed of 11.7 m/s. The particle sizes of the suspensions after milling were determined using laser diffraction, and the suspensions were refrigerated at 8 °C for a day before nanoextrusion. The same wet-milled drug suspension was used to prepare a given drug–polymer formulation with two target drug loadings: 2% and 10%; hence, identical particle sizes were reported in Table 1.

#### 2.2.2. Preparation of Extrudate Powders

**Figure 1:**
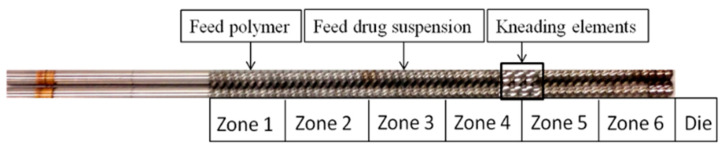
Schematic of the screw configuration and the locations of the volumetric feeder feeding the polymer powder (Zone 1) and the peristaltic pump feeding the drug nanosuspension (Zone 3).

Nanoextrusion was performed with a Process 11 co-rotating twin-screw extruder (Thermo Fisher Scientific, Waltham, MA, USA) with a die having a 1.0 mm hole. Six temperature-controlled zones were used, which were individually heated with electric heaters and cooled with water (see Figure 1). The additional dry polymer (Sol/Kol/HPC) and the wet-milled drug nanosuspension were fed to Zone 1 via a volumetric feeder and to Zone 3 via a peristaltic pump, respectively. Table 2 presents the temperatures of the individual heating zones and the die as well as the feed rates of the polymer and wet-milled suspension. The ratio of polymer and suspension feeding rates determined the final drug loading in various extrudates. Two drug loadings were produced for each polymeric matrix.

Different processing temperatures were selected based on exploratory processing experiments (not shown), previous extrusion studies with these polymers [41,65], and consideration of the glass transition temperature/melting temperature of the respective polymer and the drug, as well as potential degradation of the polymers at high temperatures. According to a TGA analysis [66], GF exhibited a slight mass decrease (2.1%) at temperatures above 220 °C due to degradation. When assayed by high performance liquid chromatography (HPLC), GF was chemically stable at 223 °C; the recovery of GF remained as high as 99.5% even after being held at that temperature for 3 h [58]. The thermal degradation temperature of Sol, as measured by TGA, is 250 °C, whereas a subtle color change in the extrudate was observed at temperatures above 170 °C, which was attributed to partial degradation of Sol [67]. While a slight mass decrease was observed above 170 °C in the TGA trace of HPC-SL, the authors claimed that HPC-SL was stable up to 250 °C [68]. On the other hand, discoloration of HPC above 195 °C was reported [59], which could imply partial degradation. The thermal degradation temperature of Kol, as measured by TGA, is 180 °C [69]. As can be seen from Table 2, the selected (maximum) extrusion temperatures in the experiments were below the degradation temperatures mentioned above; hence, the drug and the polymers are expected to be thermally stable.

We set the screw-speed at 100 rpm. Kneading elements with 60° offset angle were placed in Zones 4 and 5 (mixing zone), which imparted intense mixing and ensured homogeneous dispersion of the suspension in the molten polymer. Owing to elevated barrel temperature, most of the water evaporated and the vapor exited Zones 4–5, as the caps on the barrel were left loose. We collected the extrudates exiting the die after they cooled down to room temperature and stored them in a desiccator. As the Kol formulations had comparably low processing temperature, water evaporation was slow and residual moisture was high. To remove excess moisture, we dried the Kol formulations additionally for a day in a desiccator at room temperature. A higher processing temperature was not desirable to produce Kol threads, as Kol became a liquid with very low viscosity, leading to undesirable back-flow in the extruder as a major processing issue.

The extrudate threads were milled into powders using a coffee grinder (Model: IDS77-RB, Mr. Coffee, Cleveland, OH, USA). Drug assay, XRPD, and TGA were conducted on the milled extrudate powders. The milled powders were then passed through a series of sieves with opening sizes of 63, 125, 250, 425, and 710 µm. The milled-sieved powders/sieve fractions in the size ranges of <63 µm, 125–250 µm, and 425–710 µm were subjected to particle sizing and dissolution testing. It should be noted that throughout the paper, the extrudates were labeled with the target drug content and the polymer used. For example, 10% GF-Sol has 10% GF in the polymeric matrix of Sol.

#### 2.2.3. Measurement of Particle Size

Laser diffraction (LS 13 320, Coulter Beckman, Brea, CA, USA), with a polarized intensity differential scattering (PIDS) obscuration water optical model, was used to measure drug particle sizes in the suspensions. We maintained the PIDS between 40 and 50% and the obscuration below 8%. The Mie scattering theory was used to determine the particle size distribution. We input the refractive index values of 1.65 for GF and 1.33 for de-ionized water. About 2 mL milled suspension samples were diluted with 8 mL aq. polymer–SDS solution depending on the stabilizer(s) used in the milling experiments before the size measurement.

The particle sizes of the milled extrudate powders in the size ranges of <63 µm, 125–250 µm, and 425–710 µm were measured by a Rodos/Helos laser diffraction system (Sympatec, Pennington, NJ, USA) based on Fraunhofer theory. Approximately 1 g of the extrudate powder was placed on the sample chute of the Rodos dispersing system. As the sample chute was vibrated to feed the sample at a 50% setting, a dispersion pressure of 0.1 bar was imposed to suck in falling powder through the sample cell of the Rodos. Particle size distribution and the Sauter mean diameter *D*_32_ were computed by the instrument’s Sympatec Windox 5.0 software, which is the equipment’s own software (Sympatec, Pennington, NJ, USA). The (external) specific surface area *S*_v_ was approximately calculated using the equation: *S*_v_ = 6/*D*_32_. The extrudates before milling were in the form of coils, and their equivalent cylindrical length (equivalent to 8.9 mg and 100 mg GF dose for dissolution testing) was measured using a rope. The *S*_v_ of a single extrudate thread was calculated directly from the geometry using *S*_v_ = 4(1 + 0.5*D*/*L*)/*D*, where *D* is the diameter and *L* is the equivalent length of the extrudate thread. For example, the *D* × *L* values of the threads containing 8.9 mg dose were approximately 1 mm × ~80 mm for 10% GF-polymer and 1 mm × ~390 mm for 2% GF-polymer.

#### 2.2.4. Microscopy

A LEO 1530 SVMP (Carl Zeiss, Inc., Peabody, MA, USA) scanning electron microscope (SEM) was used to examine the morphology of the drug nanoparticles in the as-produced extrudate threads. A smooth cross-section of the thread was formed by placing it in liquid nitrogen and breaking it manually. We placed the cross-sections of the threads on an aluminum stub using carbon tape. Before imaging, a BAL-TEC MED 020 sputter coater (BAL-TEC AG, Balzers, Switzerland) was used to coat the samples with carbon.

The morphological changes of a milled extrudate particle when exposed to deionized water were visualized by an Axio Scope.A1 microscope (Carl Zeiss Microscopy, LLC, Thornwood, NY, USA). We placed a particle from the 125–250 µm fraction of the milled extrudate powders with select formulations (10% GF-Sol, 10% GF-Kol, and 10% GF-HPC) on a glass slide. Images were captured at several time points following the addition of a 3 μL drop of deionized water onto the particle.

#### 2.2.5. X-ray Powder Diffraction (XRPD)

The crystallinity of the as-received raw materials (GF and polymers), milled extrudate powders without sieving, and physical mixtures (PMs) with identical formulations to those of the extrudates were analyzed using XRPD (PANalytical, Westborough, MA, USA), provided with Cu Kα radiation (*λ* = 1.5406 Å). The samples were scanned for 2*θ* ranging from 5° to 40° at a scan rate of 0.165 s^−1^.

#### 2.2.6. Thermogravimetric Analysis (TGA)

A TGA/DSC1/SF Stare system (Mettler Toledo, Inc., Columbus, OH, USA) was used to characterize residual water via thermogravimetric analysis (TGA). We placed ~8 mg extrudate powder or as-received GF/Sol/Kol/HPC in a ceramic crucible. Under nitrogen flow, the sample was heated from 25 °C to 150 °C at a constant rate of 10 °C/min.

#### 2.2.7. Wetting of GF Powder by Soluplus^®^, Kolliphor^®^ P407, and HPC Solutions

An Attension Sigma 700 equipment (Biolin Scientific, Linthicum, MD, USA) was used to investigate GF wettability enhancement by the polymers via the modified Washburn method [70,71]. In this method, liquid penetration into a packed GF powder bed is examined, and the mass of liquid penetrated as a function of time is recorded. Supplementary Material provides all details of the experimental methods; hence, only salient features are given here. Liquids and powder refer to deionized water/stabilizer solution (Sol/Kol/HPC with SDS) and GF, respectively. The Attension Sigma 700 equipment was also used to measure surface tension of the liquids. We used an R/S Plus Rheometer (Brookfield Engineering, Middleboro, MA, USA) to measure the apparent shear viscosity. Instead of individually determining *θ*_ss_ (the contact angle between GF and the aq. solution of Sol/Kol/HPC with SDS) and *θ*_w_ (the contact angle between GF and deionized water), we calculated the ratio cos*θ*_ss_/cos*θ*_w_ using the modified Washburn method. Li et al. [41] used this ratio as a rough metric of the drug wettability enhancement upon the use of different stabilizers in water.

#### 2.2.8. GF Content in the Extrudate Powders and Their Dissolution

A previously established method [39] was used to determine the drug content in the extrudates. We dissolved 100 mg of the extrudate powders in 20 mL of methanol and sonicated it for 30 min and stored overnight to ensure that all GF particles had dissolved. A 100-μL aliquot was taken from the GF solution and diluted into 10 mL methanol. An ultraviolet (UV) spectrophotometer (Agilent, Santa Clara, CA, USA) was used to measure the absorbance of all samples at the wavelength of 292 nm. A pre-established calibration curve was then used to calculate drug concentration in the samples. The mean drug content along with the percent relative standard deviation (RSD) was calculated based on *n* = 6 samples for each formulation.

The USP II paddle method established in reference [41] was adapted here to determine GF release from the extrudates/sieved fractions using a Distek 2100C dissolution tester (North Brunswick, NJ, USA). 1000 mL deionized water, maintained at 37 °C, was stirred at a paddle speed of 50 rpm. We selected deionized water as the medium because it allows for good discrimination of different GF formulations under non-sink conditions [29,39]. In the dissolution tests, we considered two different doses of GF: 8.9 and 100 mg. We selected 8.9 mg GF dose as it could emulate potent drugs and it provides non-supersaturating conditions in water. The sample powders were poured into the dissolution medium. At various times, 4 mL samples were taken out manually. As we aim to minimize any confounding effect of undissolved coarse drug aggregates, these aliquots were filtered with a 0.1 µm PVDF membrane-type syringe filter prior to UV spectroscopy. We also used 100 mg GF dose in additional dissolution experiments to allow for generation of supersaturation. In this case, dissolution test was conducted with additional sampling for up to 210 min. Prior to UV spectroscopy; the filtered samples were diluted with 37 °C deionized water at a ratio of 1 to 7. UV spectroscopy (at a wavelength of 296 nm) was used to measure the amount of GF dissolved through a pre-established calibration curve.

To rank the normalized drug release rate from different polymeric matrices and matrix sizes under non-supersaturating conditions, fitting of GF dissolution data by Korsmeyer-Peppas model [72,73], as shown in Equation (1), was performed using the regression wizard of SigmaPlot (Version 11), which is a general-purpose graphing and statistical analysis software (Systat Software, Inc., San Jose, CA, USA).

(1) $$M_{t}/M_{\infty }=kt^{n}$$

Here, *M* is mass of the drug, *k* is a dissolution constant and *n* is the exponent; *M_t_*/*M*_∞_ is the normalized mass of the drug released at time *t*. While this model could be used to assess drug release mechanisms, it was considered here as an empirical kinetic model because several assumptions behind the mechanistic Peppas model are not satisfied; therefore, the interpretation of the release mechanism could be confounded. Fitting of *M_t_*/*M*_∞_ included data up to and including one point after attainment of *M_t_*/*M*_∞_ value of 0.60. The product *kn* exactly equals drug release rate at 1 min. As the drug release rate d(*M_t_*/*M*_∞_)/d*t* is modulated by *kn* [74]; this product *kn* was used as a simple drug release parameter to compare drug release rates from different formulations [75]. The model was not used for supersaturating dissolution conditions (100 mg GF dose) because the use of normalized drug release would be misleading for the comparison of ASDs vs. nanocomposites. Moreover, as will be shown in Section 3, the use of *kn* would not be useful because the discrimination power of the dissolution test was reduced for the nanocomposites under the supersaturation conditions; hence, impact of various formulations–matrix sizes would be harder to discern.

#### 2.2.9. Solvent-Shift Method for Study of GF Precipitation Inhibition by Polymers

The maintenance of supersaturation generated by ASDs during drug release is critical to success of such formulations during in vivo and in vitro dissolution. The effect of different polymers as precipitation inhibitors was evaluated by a solvent shift method in which drug supersaturation is generated by mixing a drug solution with an antisolvent. The solvent-shift test method was adapted from reference [76]. A concentrated solution of GF in acetone was prepared by dissolving 100 mg of as-received GF in 20 mL acetone. This solution was subsequently added to 1000 mL of 900 µg/mL aq. HPC–SDS/Sol–SDS/Kol–SDS solution, with 1:9 drug:polymer ratio and corresponding SDS in the feed formulations. This solvent-shift generated an initial GF concentration of ~100 µg/mL in the medium, which is highly supersaturated. The test was performed in a Distek 2100C dissolution tester (North Brunswick, NJ, USA). Similar to the dissolution test, the medium was maintained at 37 °C and stirred by a paddle at 50 rpm; 4 mL samples were withdrawn manually from each vessel at predefined intervals up to 210 min to assess supersaturation maintenance. These aliquots were filtered with 0.1 µm PVDF membrane-type syringe filter. Absorbance was measured using UV-vis spectrophotometer at 296 nm wavelength. All measurements were carried out in triplicate.

## 3. Results and Discussion

### 3.1. Wet Stirred Media Milling of Drug Suspensions

Table 1 presents the formulations of drug (GF) nanosuspensions and their particle sizes with standard deviation (SD) as measured by laser diffraction. In view of the as-received (unmilled) GF microparticles, i.e., *D*_10_ = 4.67 ± 0.06 μm, *D*_50_ = 14.27 ± 0.25 μm, and *D*_90_ = 37.46 ± 0.38 μm; Table 1 suggests that wet stirred media milling led to extensive breakage of GF particles down to nanoscale, which is usually regarded as 10 to few hundred nm, up to 1000 nm in the prevalent pharmaceutical literature [77]. All drug nanosuspensions have 1.9% polymer (Sol/Kol/HPC) and 0.15% surfactant (SDS) as stabilizers, respectively. The slightly higher *D*_90_ of the GF suspension with Kol and SDS could be attributed to the presence of larger GF nanoparticle aggregates. However, all the suspensions were colloidal with *D*_50_ in the range of 156–185 nm, indicating a similar drug particle size after milling despite the use of different polymeric stabilizers. Neutral polymers, such as HPC, Kol, and Sol tend to adsorb on drug particles and impart steric stabilization, whereas SDS, an anionic surfactant, when adsorbed provides electrostatic stabilization, and polymer–anionic surfactant combination could impart electrosteric stabilization [11,63,64,78]. Neutral polymer–anionic surfactant combination was shown to have synergistic stabilization of a multitude of drug nanosuspensions [28,64,79,80,81]. Specifically, HPC SL [63,64] or Sol [82] could not prevent aggregation of GF nanoparticles; hence, SDS was required along with the respective polymer to impart the necessary physical stability. A similar observation was made for drug nanosuspensions containing Kol with/without SDS [81]. On the other hand, surfactant concentration has to be optimized because high concentration of SDS, especially above the critical micelle concentration (CMC), facilitated Ostwald ripening of the particles of various drugs [64,80,83].

The positive impact of the polymer–surfactant combination could be explained by two mechanisms: (i) the steric stabilization imparted by the neutral polymer and the electrostatic stabilization imparted by the negatively charged anionic surfactant, a.k.a. electrosteric stabilization, provided that the anionic surfactant (SDS) is at sufficiently high concentration [84]; and (ii) the wettability enhancement–reduced surface tension provided by the anionic surfactant and the amphiphilic polymer [63,64,82,84]. HPC-SDS [85] and Sol-SDS [86,87] could also form clusters or complexes. These complexes may allow for co-adsorption of polymer–SDS onto drug surfaces (see e.g., [88,89]), potentially facilitating polymer adsorption [63,80]. At 0.05% *w*/*w* SDS (below CMC of 0.23% *w*/*w*), the positive impact of SDS was mainly explained by the reduced surface tension and higher wetting effectiveness factor; presence of SDS at this low concentration could not render electrostatic/electrosteric mechanism (when used along with HPC) operative or dominant [84]. Electrosteric mechanism was dominant when 0.5% *w*/*w* SDS was used along with HPC [63,64]. For 0.15% *w*/*w* SDS used in this study, we expect that both mechanisms could be operative. It is well known that lower surface tension promotes good wetting of particles in a liquid [90]. Hence, reduction in surface tension and wettability enhancement upon use of polymers/surfactants could facilitate the deaggregation of the aggregates during the wet stirred media milling [63,84]. This could also explain the synergistic action of polymer–SDS combination, as opposed to the electrosteric mechanism, because facilitated deaggregation in the presence of SDS could allow for more polymer adsorption as more drug nanoparticle surfaces are exposed to the stabilizer solution.

### 3.2. Preparation and Characterization of the Extrudates

The nanoextrusion process used the milled drug nanosuspensions and an additional extrusion polymer as two separate feeds and converted the suspension into extrudates by continuously evaporating water. As can be seen from Table 3, for the same polymer, a higher feed rate ratio of the nanosuspension to the additional polymer led to higher drug loading, and the mean drug loadings were close to the target values 2% and 10% *w*/*w*. All the produced extrudates had very low RSD, i.e., 1.0–2.2%, which signifies an advantage of nanoextrusion process in preparing uniformly distributed solid dosage forms even with low drug loading (~2%). The mean moisture content of the Sol and HPC formulations was measured immediately after the nanoextrusion, and they were relatively low (<4%) because their processing involved temperatures as high as 165 °C and 140 °C in the last two zones, respectively (refer to Table 2). To ensure proper processing and acceptable thread formation, processing temperatures were kept low for Kol formulations (maximum of 110 °C). Due to slower water evaporation at the lower temperature, the Kol-based extrudates had a moisture content of 15% for the threads of 10% GF-Kol immediately after the nanoextrusion and they were subjected to additional drying for 24 h in a desiccator. The final moisture was reported in Table 3. While nanoextrusion can be used as a continuous drier, depending on the type of drug–polymer and processing conditions, additional drying may be needed, as shown for GF–Kol.

**Figure 2:**
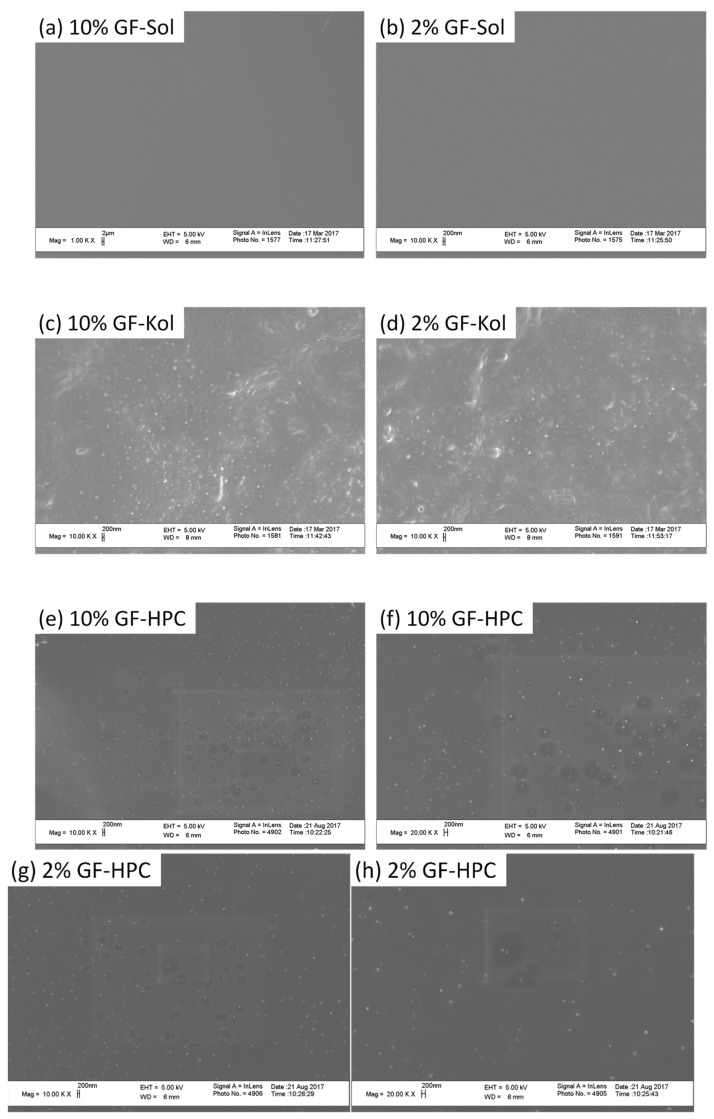
SEM images of the cross-sections of various extrudate threads: (**a**) 10% GF-Sol, (**b**) 2% GF-Sol, (**c**) 10% GF-Kol, (**d**) 2% GF-Kol, (**e**) 10% GF-HPC (10K× magnification), (**f**) 10% GF-HPC (20K× magnification), (**g**) 2% GF-HPC (10K× magnification), and (**h**) 2% GF-HPC (20K× magnification).

**Figure 3:**
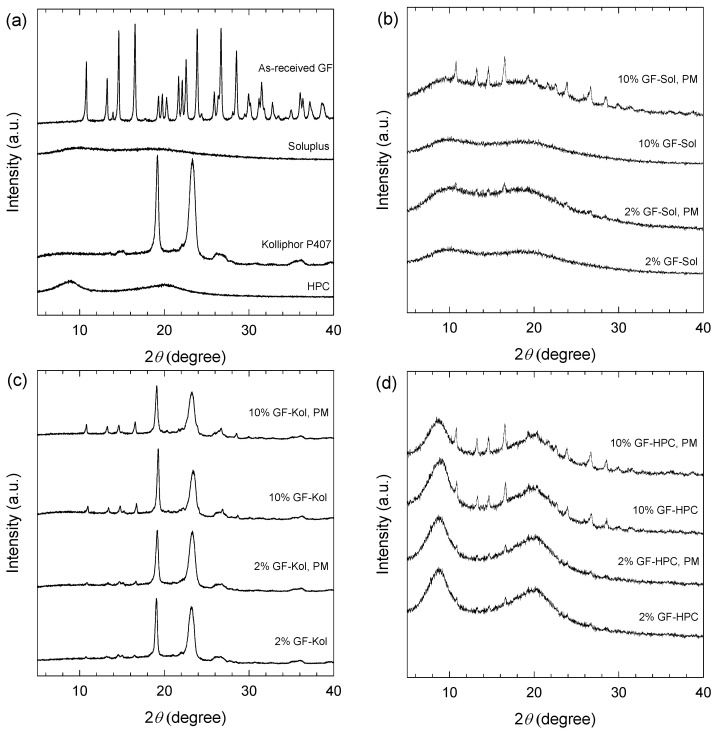
XRD diffractograms of (**a**) as-received GF particles, Soluplus^®^ (Sol), Kolliphor P407 (Kol), and HPC; (**b**) physical mixtures (PMs) and milled, unsieved extrudate powder of GF-Sol; (**c**) physical mixtures (PMs) and milled, unsieved extrudate powder of GF-Kol; and (**d**) physical mixtures (PMs) and milled, unsieved extrudate powder of GF-HPC.

The solid-state and crystallinity of the drug could impact its dissolution behavior; hence, it is important to assess the morphology and crystallinity of the drug in the produced extrudates. SEM images of the cross-section of various extrudate threads are presented in Figure 2 and corresponding XRPD diffractograms are presented in Figure 3. Since GF nanosuspensions were dried by the nanoextrusion process, the presence of GF nanoparticles in the extrudates was examined via SEM first. GF nanoparticles in the range of ~50–300 nm were embedded and dispersed in the Kol (Figure 2c,d) and HPC (Figure 2e–h) matrices as a secondary phase. Hence, these extrudates are referred to as “nanocomposites” although some minute fraction of amorphous drug may not be completely ruled out in the polymeric matrix. The presence of crystalline nanoparticles was confirmed via XRPD (see Figure 3). As-received crystalline GF microparticles and Kol, a crystalline polymer, exhibited intense characteristic crystalline peaks, whereas amorphous polymers Sol and HPC did not (Figure 3a). The physical mixture (PM) of the as-received GF particles with each as-received polymer particles exhibited similar XRPD characteristic peaks with significantly reduced intensity, which is clearly attributable to dilution and surface coverage of the GF particles by the excess polymer (Figure 3b–d). Considering that 10% GF-Kol/HPC extrudates show almost identical XRPD diffractograms to their corresponding physical mixtures (Figure 3c,d), we conclude that the GF nanoparticles in the Kol/HPC matrices were largely crystalline. Besides the dilution effect of the polymer, reduction of drug particle size to nanoscale during milling could have resulted in some XRPD peak broadening and peak height reduction [91]. It must be noted that 2% drug crystal concentration for 2% GF-Kol/HPC (Figure 3c,d) is close to the detection limit of XRPD [92,93]; while the presence of drug nanocrystals forming a secondary phase was seen from the SEM images.

Unlike the SEM images for Kol/HPC matrices (Figure 2c–h), the SEM images of Sol extrudates (Figure 2a,b) show a smooth, glassy matrix without any drug nanoparticles as a secondary phase. The XRPD diffractograms of GF-Sol extrudate did not show any characteristic GF peak (Figure 3b). Hence, both the SEM images and the XRPD diffractograms suggest that GF was molecularly dispersed within the Sol matrix, forming a single-phase amorphous mixture, i.e., an ASD.

The formation of an ASD vs. a nanocomposite is largely determined by drug–polymer interactions at the molecular level, which control the miscibility between the drug and the polymeric matrix [94,95]. The difference between the drug-polymer solubility parameters may be used as a rough guidance to estimate their miscibility. Namely, if the difference is <7.0 MPa^1/2^, they are likely to be miscible and form an ASD; if >10 MPa^1/2^, they are likely to be immiscible and formation of an ASD is unlikely [18,96]. The solubility parameters of GF, Sol, Kol, and HPC are 12.2 [97], 19.4 [69], 20.1 [69], and 24.0 [98] MPa^1/2^, respectively. The solubility parameter differences between GF-Sol, GF-Kol, and GF-HPC are calculated to be 7.2, 7.9, and 11.8 MPa^1/2^, respectively. Hence, the lower solubility parameter difference close to 7 MPa^1/2^ could explain the formation of the ASD when Soluplus^®^ was used as the extrusion polymer as opposed to HPC. Although GF-Kol has a solubility parameter difference of 7.9 MPa^1/2^, the processing temperature for GF-Kol formulations was relatively low (i.e., 110 °C at the highest, Table 2), which could explain nanocomposite formation due to lower extent of molecular mixing kinetically at the lower processing temperature. In summary, the SEM images (Figure 2) and XRPD diffractograms (Figure 3) showed that GF was amorphous in the Sol matrix, forming the ASD, and largely crystalline in the Kol and HPC matrices, forming the nanocomposites. Although a different extruder, feed rates, and only 23% GF loading in HPC/Sol were considered in our earlier study [41], the findings here about the ASD vs. nanocomposite formation were similar. Unlike [41], an in-depth and systematic examination of the impact of milling and resulting extrudate matrix size–surface area was made using multiple sieve fractions of the produced extrudates at both 2% and 10% drug loading (Section 3.3, Section 3.4 and Section 3.5).

### 3.3. Impact of Matrix Surface Area at the Non-Supersaturating Dissolution Condition

**Figure 4:**
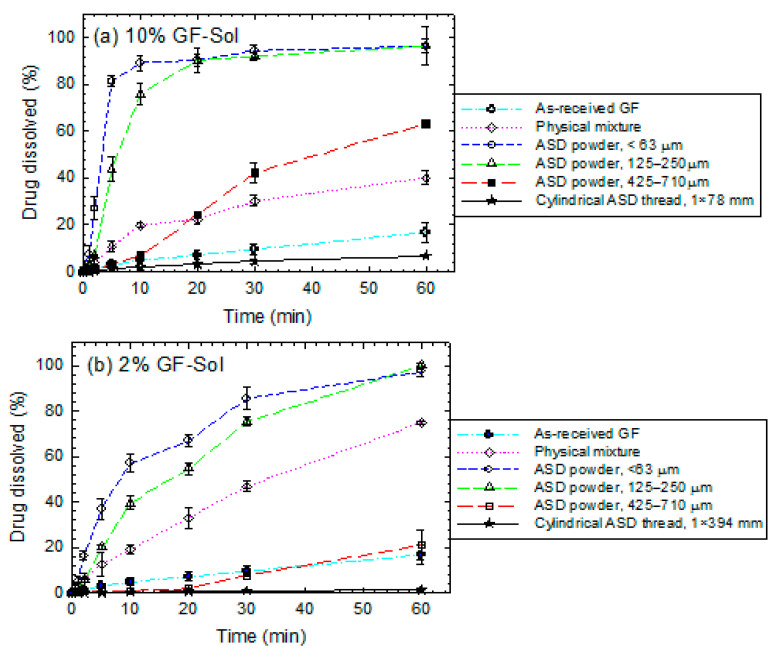

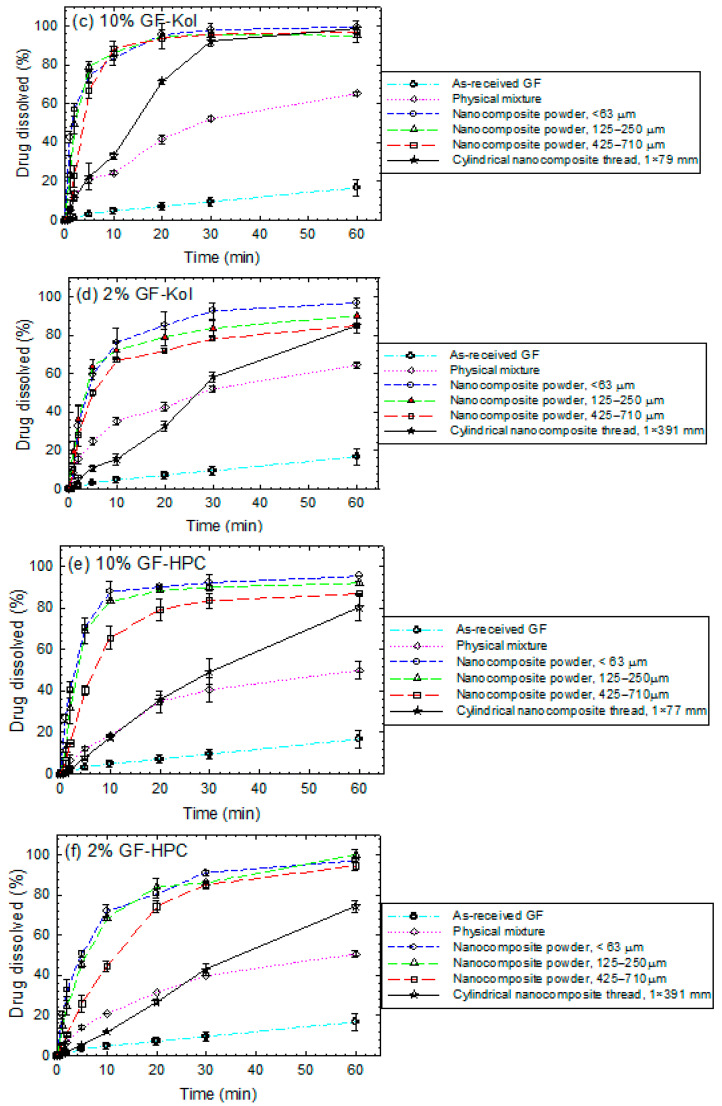
Evolution of GF release during dissolution of as-received GF microparticles, physical mixtures (PMs), thread of the extrudate, and extrudate powders with various formulations–sieved fractions: (**a**) 10% GF-Sol, (**b**) 2% GF-Sol, (**c**) 10% GF-Kol, (**d**) 2% GF-Kol, (**e**) 10% GF-HPC, and (**f**) 2% GF-HPC. The sample size is equivalent to 8.9 mg GF dose (non-supersaturating condition).

The extrudates were milled into powders and sieved into three size ranges: <63 µm, 125–250 µm, and 425–710 µm for dissolution testing. The characteristic sizes of the particles in the sieved fractions are reported in Table 3. The time-wise evolution of GF release from the unmilled extrudates (cylindrical threads), milled-sieved extrudate powders, and the physical mixtures was measured in de-ionized water using a USP II apparatus and presented in Figure 4. A relatively low drug dose of 8.9 mg was used for the dissolution tests, which will lead to an unsaturated GF solution in the dissolution medium since the solubility of GF in deionized water at 37 °C is 14.5 mg/L. The dissolution rate was quantitatively described by the drug release rate parameter (*kn*) of the Korsmeyer-Peppas model, whose fitted parameters are shown in Table 4. The majority of R^2^ values of the fittings are above 0.95, indicating a relatively good fitting by the Korsmeyer-Peppas model.

Being a BCS Class II (poorly water-soluble) drug, as-received microparticles of GF dissolved extremely slowly: <20% GF dissolved after 20 min (Figure 4). Faster GF dissolution occurred in the presence of any of the three polymers even without wet media milling-nanoextrusion (physical mixtures vs. as-received GF). We attribute this improvement to the wetting enhancement of the hydrophobic drug (GF) particles by the dissolved polymer in water, as can be seen from Table 5, which presents wetting enhancement in the presence of Sol/Kol/HPC in water; the ratio (wetting effectiveness factor) cos*θ*_ss_/cos*θ*_w_ > 1 signifies this improvement.

Let us consider the unmilled extrudates (threads) in the form of nanocomposites (Kol/HPC matrices). The large cylindrical threads with *D* × *L* of 1 mm × ~80 mm for 10% GF-polymer and 1 mm × ~390 mm for 2% GF-polymer exhibited faster GF release than as-received GF microparticles, but slower/similar release than the physical mixtures (refer to *kn* values in Table 4). Wet-milled drug particles in the nanocomposite threads dissolved faster than the as-received GF microparticles, although the large Kol/HPC matrix of the cylindrical threads took much more time to erode and release the drug nanoparticles as compared with the milled extrudate powders. Most interestingly, the cylindrical thread of GF-Sol (ASD) exhibited slower GF dissolution than the as-received GF microparticles (unprocessed). While GF was amorphous in the GF-Sol extrudates (refer to Section 3.2), the expected dissolution enhancement from the amorphous GF was not achieved under the non-supersaturating condition: only 6.8 ± 0.31% GF dissolved in 60 min from the 10% GF-Sol thread, and lower for the 2% GF-Sol thread. Without delving into details, these results clearly demonstrate the necessity for milling the extrudates, especially the ASD extrudates with Sol matrix.

Analysis of the dissolution profiles of the milled/sieved extrudates (powders) in Figure 4 and *kn* values in Table 4 suggests the following general trends: (i) smaller extrudate particles (higher specific surface area) led to faster drug release for all formulations, suggesting the importance of dry milling of the extrudates; (ii) higher drug loading (10% vs. 2%) accompanied with a reduction of polymer loading achieved faster drug release; (iii) the GF-Sol ASD exhibited a slower drug release and a markedly stronger dependence on the matrix size than the nanocomposites (GF-Kol/HPC); and (iv) at 10% GF loading, the cumulative drug release curves of <63 µm size fraction did not show a notable difference after the initial few minutes for the three different polymers used. Regarding (iv), it seems that when *kn* values are above a certain high value (e.g., >~12%min^−*n*^), their differences may not imply significant differences in the cumulative drug release after the initial few minutes. However, this does not invalidate the generality of the trend (iii).

**Figure 5:**
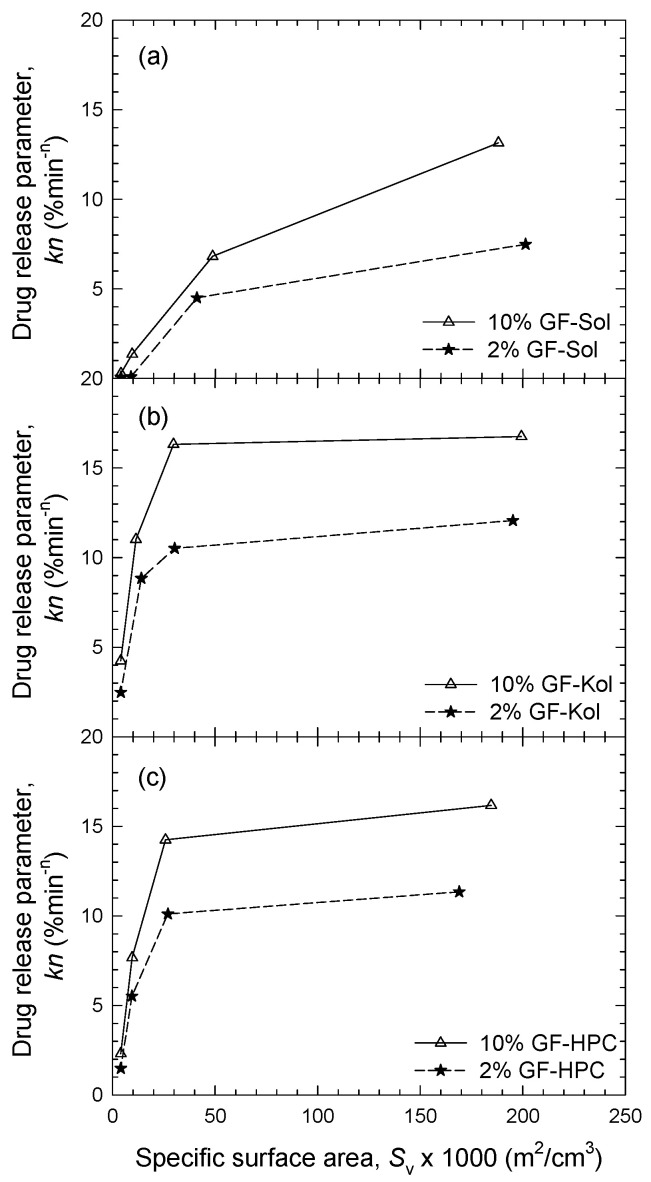
The drug release parameter *kn* as a function of the specific surface area *S*_v_ of the thread of the extrudate and the extrudate powders with various formulations–sieve fractions: (**a**) GF-Sol, (**b**) GF-Kol, and (**c**) 10% GF-HPC. The sample size is equivalent to 8.9 mg GF dose in the dissolution experiment (non-supersaturating condition).

Figure 5 illustrates a surprising and qualitatively different dependence of the drug release parameter (*kn*) on the external specific surface area *S*_v_ of the ASD (GF-Sol) vs. the two different types of nanocomposite particles (GF-Kol/HPC). While the milling of the extrudates increased the GF release rate for the nanocomposites, this effect tends to saturate above ~30 × 10^−3^ m^2^/cm^3^ for both Kol and HPC matrices at two drug loadings (Figure 5b,c). In contrast, for the ASD (GF-Sol), this positive impact was a monotone increasing function of *S*_v_ up to the highest *S*_v_: 190 × 10^−3^ m^2^/cm^3^ (10% GF) and ~210 × 10^−3^ m^2^/cm^3^ (2% GF) (Figure 5a).

A direct comparison of the dissolution performances of the ASD and the nanocomposites allows us to analyze the impact of the GF physical state (amorphous vs. nanocrystalline) and the polymeric matrix type (refer to Figure 4 and Table 4 for *kn* values). The Sol extrudate had amorphous GF (refer to Figure 3b), whereas Kol and HPC extrudates had largely nanocrystalline GF (Figure 3c,d). It is well known that crystalline drug nanoparticles in the nanocomposites have lower apparent drug solubility than the amorphous drug in the ASD. Therefore, we would expect that the ASD should outperform the nanocomposites. Surprisingly, the crystalline GF nanoparticles in the nanocomposites led to faster GF release than the amorphous GF in the ASD under the non-supersaturating dissolution condition (8.9 mg dose). The *kn* values in Table 4 also confirm the rank-order of the rate of drug release from the different polymeric matrices: Kol > HPC > Sol for all matrix sizes and both 2% and 10% drug loadings. However, we note that 10% GF loaded extrudate powders within the < 63 µm sieved fraction did not exhibit notable differences in the cumulative drug released after the initial few minutes. This is expected from the qualitative behavior of the milled extrudates and sensitivity of drug release from the ASD powder to the specific surface area (refer to Figure 5). Only for the < 63 µm sieved fraction at 10% GF loading, the dissolution advantage of the nanocomposites over the ASD for the low drug dose was not discernible after few minutes into the dissolution.

**Figure 6:**
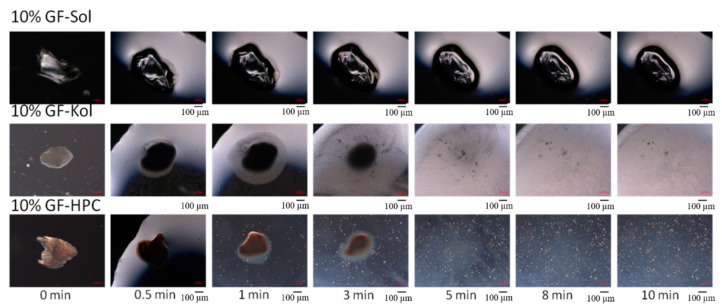
Digital microscope images showing the morphological evolution of a 10% GF-Sol (ASD) particle, a 10% GF-Kol (nanocomposite) particle, and a 10% GF-HPC (nanocomposite) particle in 3 µL deionized water. Scale bar is identical for all images: 100 µm.

The observed polymeric matrix effect may be elucidated by the interaction of an extrudate particle with water. Figure 6 illustrates the morphological changes that occurred when we added a water droplet onto a milled extrudate particle (125–250 µm fraction). The polymer dissolved upon wetting, and the Kol and HPC matrices disappeared, releasing drug nanoparticles immediately when the particle was exposed to water. The situation was quite different for the Sol matrix: it maintained its shape without significant erosion or disintegration, which suggests the amorphous GF must dissolve in the matrix and diffuse through it before being released into the dissolution medium. Since the diffusion distance is proportional to the matrix (particle) size, this observation could explain the significant dependence of GF release on the matrix size/surface area for the Sol-based ASD (refer to Figure 5a). This finding may also rationalize why the dissolution curves were not significantly different for the <63 µm fraction of 10% GF loaded extrudates after the first few minutes; the diffusion distance for the ASD was much smaller for the <63 µm fraction than those for the 125–250 µm and 425–710 µm fractions.

Besides the optical microscope imaging, the study of the wettability enhancement of GF by the polymeric matrix could shed additional light. The calculated wetting effectiveness factors, i.e., cos*θ*_ss_/cos*θ*_w_, for the aqueous solutions of Sol, Kol, and HPC penetrating a packed GF powder bed were 1.86, 14.6, and 22.2, respectively (refer to Table 5). The wettability enhancement of the polymers can be rank ordered as follows: HPC > Kol >> Sol. It is noted that cos*θ*_ss_/cos*θ*_w_ is inversely related to the hydrophobicity of the polymer, which will be discussed in Section 3.4. The fast redispersion and release of the drug nanoparticles observed in Figure 6 could be attributed to the higher cos*θ*_ss_/cos*θ*_w_ imparted by HPC and Kol, which could explain the associated immediate drug release in the dissolution testing. Besides drug wettability enhancement, the molecular weight and polymer dissolution rate could also play a role in the dissolution behavior. Besides imparting high wettability enhancement, Kol has the lowest molecular weight (MW = 9840–14,600 g/mol) as compared with HPC-SL (MW = ~100,000 g/mol) and Soluplus (MW = ~118,000 g/mol) and dissolved–eroded the fastest, thus enabling the fastest GF nanoparticle redispersion and its dissolution. Even though the GF-Sol ASD has high supersaturating capability, under the non-supersaturating dissolution condition (low drug dose), the dissolution enhancement appears to be mainly controlled by the hydrophobicity and molecular weight of the polymeric matrix and its erosion as well as the surface area of the respective polymeric matrix. Only when the <63 µm fraction of the 10% GF loaded extrudates was used, which would be akin to fine, aggressive milling of the extrudates, the differing hydrophobicity and molecular weight impact of the respective polymers became less discernible, and the ASD and the nanocomposites performed similarly after few minutes into the dissolution.

### 3.4. Impact of Matrix Surface Area at the Supersaturating Dissolution Condition

**Figure 7:**
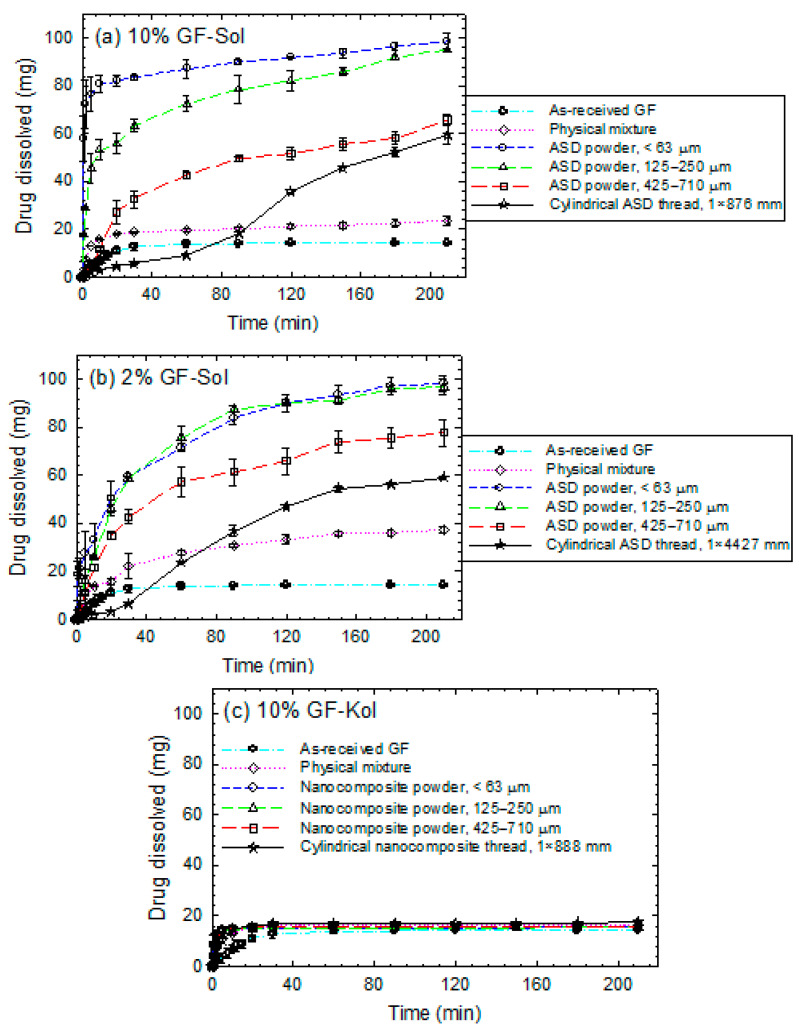

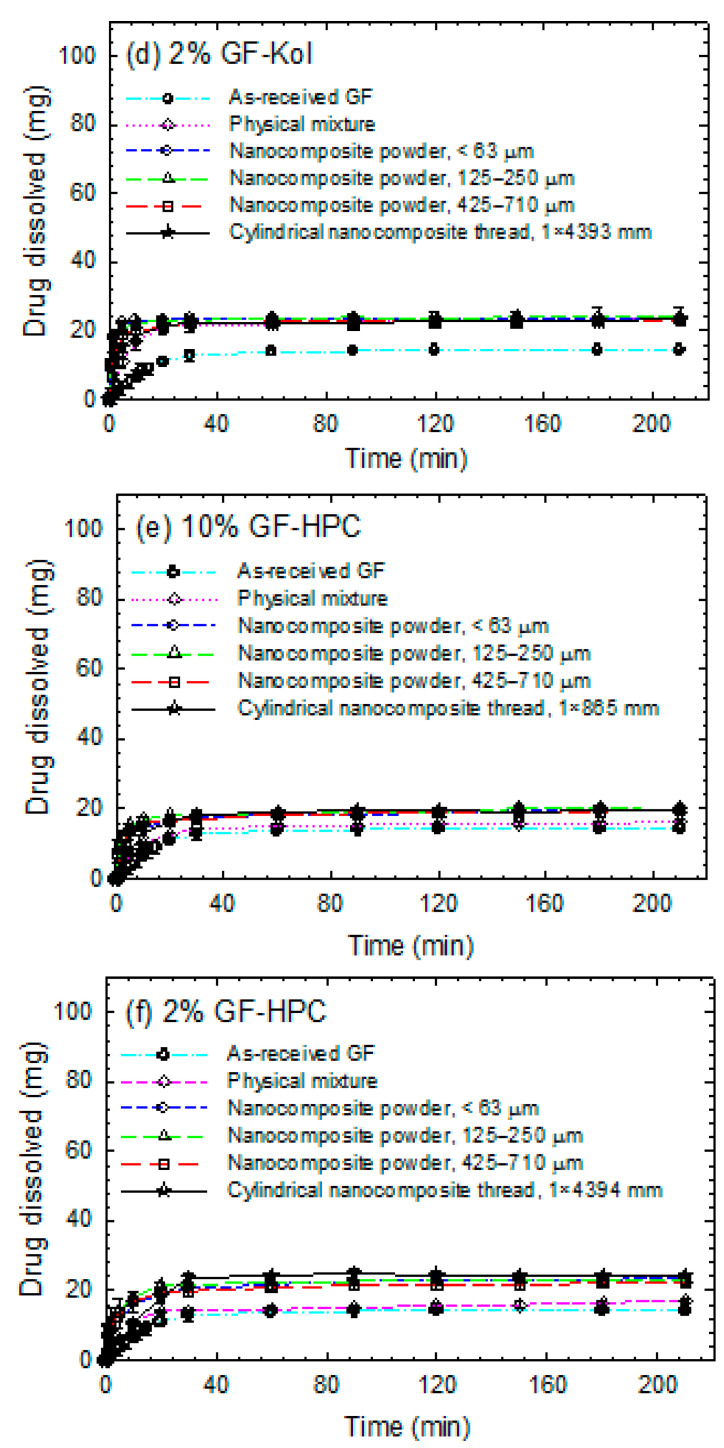
Evolution of GF release during dissolution of as-received GF microparticles, physical mixtures (PMs), thread of the extrudate, and extrudate powders with various formulations–sieve fractions: (**a**) 10% GF-Sol, (**b**) 2% GF-Sol, (**c**) 10% GF-Kol, (**d**) 2% GF-Kol, (**e**) 10% GF-HPC, and (**f**) 2% GF-HPC. The sample size is equivalent to 100 mg GF dose (supersaturating condition).

The dissolution testing was conducted with an equivalent of 100 mg GF dose, which could lead to supersaturation, i.e., drug release above 14.5 ± 0.3 mg in the dissolution medium. Figure 7 presents the actual amount of GF released, which is equivalent to % drug release because 100 mg GF dose was used. The smaller ASD particles with higher surface area led to significantly faster GF release from the Sol matrix and >80% GF release was achieved at 120 min for the <63 µm and 125–250 µm fractions (Figure 7a,b). Nearly complete drug release occurred at 210 min for the same fractions. Only 10% GF ASD with 190 × 10^−3^ m^2^/cm^3^ surface area (<63 µm fraction) achieved immediate release (80% release within 30 min). Overall, the ASD powders exhibited strong surface area dependence, thus requiring a fine milling for fast/immediate drug release. Unlike for the ASDs, the matrix size/surface area impact does not seem to be remarkable for the nanocomposites because the observed lower extent of GF dissolution was mostly controlled by the lower thermodynamic solubility of the nanocrystalline drug itself (Figure 7c–f).

Our findings agree well with the findings from a recent study by Zhang et al. [56], wherein 25% proprietary drug-loaded HPMCAS- (hydroxypropyl methylcellulose acetate succinate, MF grade) based HME extrudates (ASD) were milled via Fitz-milling, ball milling, and cryomilling to produce powders with a mean size of 213, 59, and 16 µm, respectively. The dissolution tests reveal that cryomilling led to the fastest drug release. Similarly, Zheng et al. [57] demonstrated an increase in the dissolution rate and achievement of higher supersaturation from a melt-quenched GF-Kollidon VA 64 ASD upon an increase in the extent of size reduction and polymer loading. Maximum GF release at 120 min was ~40%, ~46%, and ~56% when milled powders with 250–355 µm, 125–250 µm, and 44–75 µm size fractions were used, respectively. Unlike in this study, in reference [57], immediate GF release and full dissolution of 50 mg GF dose were not achieved from the GF ASDs due to high (50%) GF loading and use of Kollidon VA64, which is less effective as a precipitation inhibitor of GF than Soluplus [24] (identical dissolution conditions used in both Zheng et al. [57] and the current study).

While full release of the drug is important for achieving a desired pharmacokinetic profile and ensuring complete utilization of the drug dose, immediate release (80% drug released in 30 min) is not a requirement for solid dosages containing poorly soluble drugs and it may not even be achievable (e.g., [57,99]), and/or desirable except for therapeutic indications where a rapid onset of action is required, for example, pain management [100]. In this study, although GF is an antifungal drug, immediate release from the 10% GF loaded, milled extrudates was regarded to be “desirable” within the context that a higher GF loading could cause a significant dissolution slow-down [57] (see also Section 3.5). Although more work on the elucidation of the matrix polymer–size effects is needed for ASDs, in view of references [56,57] and this work, we conclude that milling is a critical process of any fusion-based or extrusion-based process train in the manufacturing of ASDs. It is worth mentioning that neither reference [56] nor reference [57] used nanoextrusion and various polymers with different drug–polymer miscibility to prepare both ASDs and nanocomposites and compared them.

Figure 7 clearly illustrates a marked difference in drug release from the ASD (GF-Sol) vs. the nanocomposites (GF-Kol/HPC). The ASD exhibited a much higher extent of GF release (up to full dissolution of 100 mg GF) than the nanocomposites for both drug loadings in the extrudates. For example, 98.8 ± 3.5 mg, 16.5 ± 0.4 mg, and 16.3 ± 0.5 mg of GF dissolved in 210 min from the extrudate powders with Sol, Kol, and HPC matrices, respectively, with 10% drug loading and <63 µm size fraction. The nanocomposites containing GF nanocrystals supersaturated slightly and attained a steady state within 30 min, and the drug release was mostly limited/controlled by the crystalline solubility of the drug, i.e., 14.5 ± 0.3 mg. The slight supersaturation could be due to the presence of polymer–SDS in the dissolution medium, the Gibbs–Thomson effect associated with high curvature of drug nanoparticles [101], and a small quantity of amorphous GF that cannot be detected by XRPD.

The drastically different dissolution behavior between the ASD and the nanocomposites with high drug dose is expected from basic thermodynamic considerations [102,103]: when used at a dose above the thermodynamic solubility limit, the amorphous form of the drug (GF–Sol ASD) attained higher supersaturation and exhibited higher extent/amount of drug dissolution than the nanocrystalline form of the drug in the nanocomposites (Figure 7c–f). Rahman et al. [24] suggested that GF solubilization inside Sol micelles with ~80 nm z-average size could also contribute to the dissolution enhancement besides the increased kinetic solubility of the drug as the concentration of Sol in the dissolution medium was estimated to be much higher (~900 mg/L) than its CMC value of 7.6 mg/L [104]. Micellar solubilization coupled with the reduced particle size contributes to the faster and higher extent of the GF release from the Sol-based ASDs [105]. Formation of a Sol–SDS complex was not likely as the estimated SDS concentration in the dissolution medium was extremely small (~0.7 mg/L) as compared with the CMC of SDS (2.3 g/L). Unlike HPC, Kol forms micelles at ~900 mg/L; however, GF did not appear to be dissolved in the Kol micelles based on the dissolution results (Figure 7c,d) or such micelles have sizes greater than 100 nm and filtered out prior to the dissolution tests.

**Figure 8:**
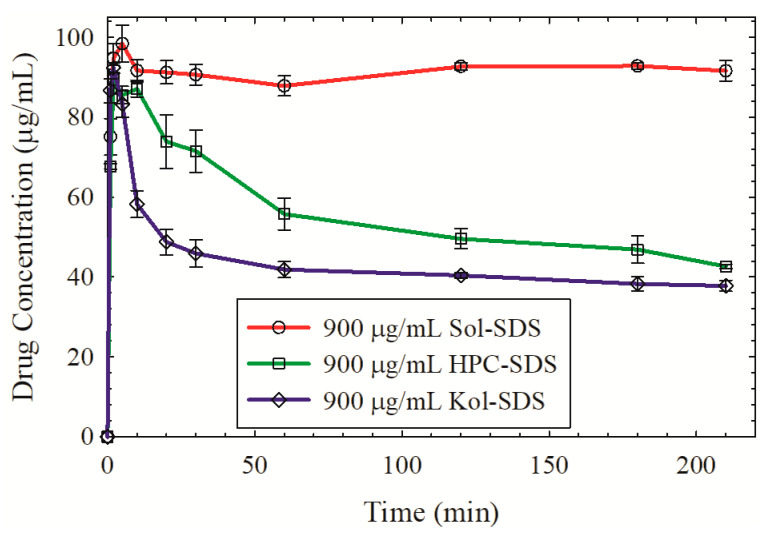
Effects of polymer–SDS on the precipitation inhibition from supersaturated solutions of griseofulvin with an initial concentration of ~100 µg/mL. The test fluid contains 900 µg/mL of polymer–SDS equivalent to 10% GF–polymer formulations. Error bar represents the standard deviation (*n* = 3).

As can be seen from Figure 8, Sol is superior to Kol and HPC in preventing precipitation of GF from a supersaturated solution. Hence, the Sol-based ASD outperformed the Kol- and HPC-based nanocomposites as Sol allows for GF ASD formation and acts as a good precipitation inhibitor during the dissolution in a supersaturated solution. There are three major factors that could explain why Sol is a better precipitation inhibitor of GF than HPC/Kol: (i) Sol lowered the driving force for GF nucleation–crystal growth due to micellar solubilization of GF molecules in the Sol micelles [24], which reduces the concentration of the free GF molecules available for nucleation–growth and the “true supersaturation”; (ii) stronger intermolecular interactions between Sol and GF as compared with those between HPC/Kol and GF; and (iii) Sol is more hydrophobic than HPC/Kol.

Precipitation of molecules from a supersaturated solution proceeds through two-steps: nucleation and growth of the nuclei [22]. Polymers with higher hydrophilicity were found to have a smaller impact on delaying nucleation than those with higher hydrophobicity/amphiphilicity [106,107,108]. Sol is a triblock graft copolymer consisting of polyethylene glycol (13% PEG 6000), polyvinyl caprolactam (57%), and polyvinyl acetate (30%) [109]. It is an amphiphilic polymer, wherein PEG provides hydrophilicity, whereas vinyl caprolactam and vinyl acetate domains are hydrophobic. While Kol (P407) is also amphiphilic and regarded as a “polymeric surfactant” with micelle forming ability, it is more hydrophilic than Sol, as signified by the higher hydrophilic lipophilic balance (HLB) value: ~22 [110] vs. 14 [109]. From a different perspective, the solubility parameter provides a numerical estimate of the intermolecular forces within a material and can be a good indication of solubility for polymers [111]. The higher the solubility parameter of a polymer, the more hydrophilic it is [112]. The solubility parameters of Sol, Kol, and HPC are 19.4 [69], 20.1 [69], and 24.0 [98] MPa^1/2^, respectively. Hence, the hydrophobicity of the polymers is ranked as follows: Sol > Kol > HPC. Besides the higher hydrophobicity of the polymer, which helps to delay nucleation, good compatibility/miscibility of the drug and the polymer is another indicator for the nucleation inhibition capability of the polymers [113,114,115]. The solubility parameter differences between GF-Sol, GF-Kol, and GF-HPC are 7.2, 7.9, and 11.8 MPa^1/2^, respectively. Therefore, the solubility parameter difference also suggests that Sol is a better nucleation inhibitor than Kol/HPC.

Kol/HPC was not a good nucleation inhibitor of GF; fast GF desupersaturation occurred via nucleation almost instantaneously after high supersaturation was attained within a few minutes (Figure 8). On the other hand, Kol seems to inhibit crystal growth better than HPC, as signified by the slower desupersaturation after 10 min. Ilevbare et al. [112] studied seeded crystallization of ritonavir as a model poorly water-soluble drug by adding seed drug crystals to supersaturated drug solutions and monitoring bulk crystal growth rates. The extent of polymer adsorption on a drug crystal surface is likely affected by the polymer hydrophobicity, which in turn affects the blockage of the sites on the crystal surface while new growth units or drug molecules are integrated into the crystal surface [112]. Their extensive systematic analysis revealed that moderately hydrophobic polymers within the solubility parameter range of 20.56–25.98 MPa^1/2^ were effective crystal growth inhibitors, albeit to varying extents, while more hydrophobic polymers with a solubility parameter <20.56 MPa^1/2^ were ineffective growth inhibitors. This finding could explain why HPC could be a better growth inhibitor than Kol, and it could also imply that although Sol is a good nucleation inhibitor, it may not be a good growth inhibitor (e.g., in the case of significant amount of residual crystals present in the ASD extrudate).

### 3.5. The Interplay between Matrix Size and Drug Loading at Supersaturating Dissolution Condition

In the low-dose GF case (Figure 5), the extrudates with 10% GF loading exhibited faster GF release than those with 2% GF loading. In the high dose case (Figure 7a,b), 10% GF-Sol dissolved faster than 2% GF-Sol for the < 63 µm size fraction; however, above 425 µm, 2% GF-Sol dissolved faster than 10% GF-Sol. This anomalous behavior may signify a complex interplay between the drug loading and the particle size/specific surface area in their combined effect on the drug release for high dose ASDs. Indeed, in our previous study [41], a 23.5% drug loaded milled GF-Sol extrudate with 125–250 µm size fraction (*D*_50_ = 122 ± 9.0 µm, *D*_90_ = 203 ± 13 µm) and 100 mg dose released 35, 43, and 62 mg GF at 20, 30, and 120 min, respectively. Here, at identical dissolution conditions with 100 mg GF dose, the 10% drug loaded milled GF–Sol extrudate with even slightly higher *D*_50_ and similar *D*_90_ (refer to Table 3 for the 125–250 µm size fraction) released 56, 64, and 83 mg GF at the same time points, respectively. Hence, we find that an increase in the drug loading from 10% to 23.4% led to a significantly slower GF release. This analysis suggests that a higher drug loading than 10% could entail more aggressive and/or prolonged milling, yielding finer extrudate particles with a higher specific surface area for rapid drug release. Hence, the interplay among drug loading–specific surface area–drug release warrants further elucidation by studying several drug loadings above 10% with various extrudate sizes.

## 4. Conclusions

This study has examined the role of milling and resulting matrix size/surface area in GF release during the dissolution of Kol/HPC/Sol-based extrudates and elucidated different milling requirements for ASDs vs. nanocomposites prepared via nanoextrusion. Due to differences in polymer–drug miscibility and processing temperatures, nanoextrusion of GF-Sol lead to an amorphous solid dispersion (ASD), whereas that of GF-Kol/HPC led to nanocomposites. Under the *non-supersaturating dissolution condition* (low GF dose), the higher specific surface area, and 10% vs. 2% drug loading led to a more rapid GF release, signified by the higher values of the dissolution rate parameter. For the nanocomposites, when the milled matrix had a specific surface area above 30 × 10^−3^ m^2^/cm^3^, the impact on dissolution became less significant. In contrast, the ASD exhibited a remarkably stronger matrix surface area dependence of drug release compared to the nanocomposites. The nanocomposites dissolved faster than the ASD due to fast eroding/dissolving matrix of Kol/HPC as compared with the slower drug release from the slowly eroding/dissolving Sol matrix. However, the cumulative drug release after a few minutes into the dissolution did not exhibit much difference for the 10% GF loaded extrudate powders with < 63 µm sieve fraction.

Under the *supersaturating dissolution condition* (high drug dose), the ASD outperformed the nanocomposites in terms of higher extent of drug dissolution as amorphous GF allowed for supersaturation while Sol enabled micellar solubilization and maintained the high supersaturation as a good precipitation inhibitor owing to its higher hydrophobicity as compared with HPC/Kol. The nanocomposites did not exhibit significant supersaturation. For the ASD, the matrix surface area appeared to play a predominant role in GF release. Only 10% GF ASD with 190 × 10^−3^ m^2^/cm^3^ surface area achieved immediate release (80% release within 30 min). Overall, this study suggests the critical importance of milling in modulating drug release from ASDs and the need for fine milling that yields a specific surface area >~200 × 10^−3^ m^2^/cm^3^ for rapid drug release, whereas only a coarse milling yielding ~30 × 10^−3^ m^2^/cm^3^ surface area could enable nanocomposites to release low-dose drugs rapidly. Finally, this study also implies a complex interplay among the drug release, the drug loading (2%, 10%, and potentially higher drug loading), and the specific surface area of the milled ASDs: a finer milling/higher specific area is required to achieve faster GF release from the ASDs with >10% drug loading, which will be thoroughly and systematically investigated in a future study.

## Figures and Tables

**Table 1:** Formulations and particle sizes of the drug (GF) nanosuspensions fed to the extruder during the nanoextrusion experiments.

Formulation ID	Polymer ^a^	Drug Particle Size after Milling (nm)		
		*D*_10_ ± SD	*D*_50_ ± SD	*D*_90_ ± SD
10% GF-Sol	Soluplus^®^	113 ± 0	156 ± 1	228 ± 1
2% GF-Sol	Soluplus^®^	113 ± 0	156 ± 1	228 ± 1
10% GF-Kol	Kolliphor^®^ P407	100 ± 8	185 ± 4	358 ± 49
2% GF-Kol	Kolliphor^®^ P407	100 ± 8	185 ± 4	358 ± 49
10% GF-HPC	HPC	121 ± 1	159 ± 0	215 ± 1
2% GF-HPC	HPC	121 ± 1	159 ± 0	215 ± 1

^a^ Extrudates with any given polymer type used the same drug nanosuspension as the feed.

**Table 2:** Process parameters used in the nanoextrusion experiments for various formulations.

Formulation ID	Polymer	Feeding Rate (g/min)		Processing Temperature (°C)							Residence Time (s)
		Polymer ^a^	Suspension ^b^	Zone 1	Zone 2	Zone 3	Zone 4	Zone 5	Zone 6	Die	
10% GF-Sol	Soluplus^®^	2.9	1.4	70	70	100	100	165	165	165	120
2% GF-Sol	Soluplus^®^	2.7	0.25	70	70	90	100	165	165	165	115
10% GF-Kol	Kolliphor^®^ P407	2.9	1.4	10	25	100	100	110	110	90	120
2% GF-Kol	Kolliphor^®^ P407	2.8	0.28	10	25	95	100	110	110	85	110
10% GF-HPC	HPC	2.8	1.4	70	120	120	140	140	140	140	116
2% GF-HPC	HPC	2.8	0.27	70	120	120	140	140	140	140	120

^a^ Refers to the additional polymer mixed with the drug nanosuspension during the nanoextrusion process. ^b^ All milled drug suspensions have 22.6% *w/w* GF, 1.9% polymer, and 0.15% SDS for all formulations.

**Table 3:** Drug–moisture content of the extrudates and characteristic particle sizes of three sieve fractions obtained from the milling–sieving of the extrudates.

Formulation ID	Drug Content (RSD) (% *w/w*) ^a^	Moisture Content ± SD (% *w/w*) ^a,b^	<63 µm			125–250 µm			425–710 µm		
			*D*_10_ (µm), SD (µm)	*D*_50_ (µm), SD (µm)	*D*_90_ (µm), SD (µm)	*D*_10_ (µm), SD (µm)	*D*_50_ (µm), SD (µm)	*D*_90_ (µm), SD (µm)	*D*_10_ (µm), SD (µm)	*D*_50_ (µm), SD (µm)	*D*_90_ (µm), SD (µm)
10% GF-Sol	9.8 (1.5)	3.6 ± 0.0	12.9, 0.1	51.7, 0.1	104, 0.2	104, 1.6	146, 0.8	195, 3.7	469, 17	716, 20	845, 3.5
2% GF-Sol	1.9 (1.9)	3.2 ± 0.5	13.7, 0.3	42.8, 0.6	90.7, 0.5	104, 1.1	158, 2.5	230, 8.2	480, 20	726, 32	847, 4.3
10% GF-Kol	9.7 (2.2)	0.8 ± 0.2 ^c^	12.7, 3.4	40.0, 2.3	81.0, 9.9	123, 17	195, 9.9	278, 6.8	371, 7.7	497, 29	581, 14
2% GF-Kol	2.1 (1.9)	0.7 ± 0.1 ^c^	17.1, 0.9	39.6, 1.2	67.5, 6.7	138, 2.3	210, 8.7	280, 3.4	380, 11	492, 24	604, 37
10% GF-HPC	10.3 (1.0)	2.9 ± 0.2	16.9, 1.7	43.9, 9.1	115, 7.6	126, 3.3	195, 5.1	294, 16	464, 21	634, 17	812, 8.1
2% GF-HPC	2.1 (1.4)	2.8 ± 0.2	21.3, 1.4	57.1, 0.6	123, 19	127, 2.4	185, 8.8	266, 16	446, 36	616, 33	799, 21

^a^*w*/*w* with respect to total extrudate mass. ^b^ Moisture content of raw materials: GF: 0.2%, Sol: 2.4%, Kol: 0.3%, and HPC: 2.6%. ^c^ Extrudates additionally dried in a desiccator for 24 h.

**Table 4:** Statistical analysis of the Korsmeyer–Peppas model fit to the drug release profiles of extrudates (thread and powders) with various polymers and size fractions (8.9 mg GF dose).

Formulation ID	Specification of Size (µm) ^a^	Korsmeyer-Peppas Model ^a^			*kn* (%min^−^*^n^*)
		*n* (-)	*k* (%min^−*n*^)	R^2^ (-)	
As-received GF	–	0.746	0.785	0.992	0.58
10% GF-Sol	PM	0.530	4.72	0.965	2.50
	<63	1.30	10.1	0.991	13.2
	125–250	1.10	6.19	0.953	6.81
	425–710	0.932	1.46	0.963	1.36
	Thread	0.697	0.40	0.988	0.28
2% GF-Sol	PM	0.737	3.69	0.999	2.72
	<63	0.568	13.2	0.950	7.86
	125–250	0.745	6.04	0.979	4.50
	425–710	1.43	0.06	0.989	0.09
	Thread	0.628	0.11	0.948	0.07
10% GF-Kol	PM	0.492	9.06	0.976	4.46
	<63	0.389	43.1	0.981	16.8
	125–250	0.527	31.0	0.939	16.3
	425–710	1.27	8.66	0.990	11.0
	Thread	0.903	4.70	0.984	4.24
2% GF-Kol	PM	0.406	12.7	0.981	5.14
	<63	0.616	19.6	0.900	12.1
	125–250	0.355	29.6	0.939	10.5
	425–710	0.376	23.5	0.934	8.84
	Thread	0.824	3.01	0.981	2.48
10% GF-HPC	PM	0.546	5.76	0.953	3.14
	<63	0.603	26.8	1.000	16.2
	125–250	0.963	14.8	0.978	14.2
	425–710	0.894	8.59	0.977	7.67
	Thread	0.826	2.80	0.991	2.31
2% GF-HPC	PM	0.528	6.11	0.981	3.23
	<63	0.521	21.8	0.995	11.3
	125–250	0.641	15.8	0.997	10.1
	425–710	0.795	6.95	0.997	5.52
	Thread	0.945	1.58	0.994	1.49

^a^ PM: physical mixture of GF–polymer powder; thread refers to the unmilled extrudate obtained from nanoextrusion without further milling–sieving.

**Table 5:** Properties of various aqueous stabilizer solutions and the wetting effectiveness factor calculated from their penetration into packed GF powder during the Washburn experiment.

Liquid ^a^	Slope, (g^2^/s)	R^2^ (-)	*η* (cP)	*ρ* (g/mL)	*γ* (mN/m)	cos*θ*_ss_/cos*θ*_w_ (-)
Water	7.00 × 10^−3^	0.990	0.890	1.00	66.5	1
Aq. Soluplus^®^	6.00 × 10^−4^	0.993	12.0	1.01	40.6	1.86
Aq. Kolliphor	1.70 × 10^−3^	0.999	29.5	1.01	35.9	14.6
Aq. HPC	2.00 × 10^−4^	0.999	403	1.02	37.9	22.2

^a^ Refer to Supplementary Materials for details.

## Data Availability

Data are contained within the article and Supplementary Materials.

## Supplementary Materials

The following are available online at https://www.mdpi.com/article/10.3390/pharmaceutics13071036/s1, Figure S1: Temporal evolution of the liquid mass penetrated a packed bed of as-received GF particles by various aqueous stabilizer solutions and deionized (DI) water; Section S.1. Details of the characterization methods used for drug wettability measurements.
