# Supplementary material for: Impact of Matrix Surface Area on Griseofulvin Release from Extrudates Prepared via Nanoextrusion
Source: Pharmaceutics. 2021 Jul 7;13(7):1036. doi: 10.3390/pharmaceutics13071036 (PMC8308970; doi:10.3390/pharmaceutics13071036)
Supplement: Supplementary file 1 [file pharmaceutics-13-01036-s001.zip › pharmaceutics-1261250-SI.pdf]

pharmaceutics

Supplementary Materials: Impact of Matrix Surface Area on Griseofulvin Release from Extrudates Prepared via Nanoextrusion

Meng Li, Casey Furey, Jeffrey Skros, Olivia Xu, Mahbubur Rahman, Mohammad Azad, Rajesh Dave and Ecevit Bilgili

Section S.1.Details of the characterization methods used for drug wettability measurements

Penetration of a liquid into a packed powder bed of a drug inside a cylindrical column allows for measurement of the drug powder wettability, based on the modified Washburn method [1,2]. In the current study, liquids and powder are aqueous stabilizer solutions of Soluplus®/Kolliphor®/HPC with SDS and GF, respectively. All solutions had 15% polymer–0.07% SDS. The solutions and deionized water were saturated with griseofulvin and stirred overnight. After overnight stirring, the undissolved drug was separated from the solutions/water. All percentages are w/w with respective to deionized water. These concentrations were selected so that the viscosity can be accurately measured in our viscometer set-up. The apparent shear viscosity and surface tension of the solutions were measured using R/S Plus Rheometer (Brookfield Engineering, Middleboro, MA, USA) and Attension Sigma 700 (Biolin Scientific, Linthicum, MD, USA) respectively, as described below; then, the drug wettability was quantified via a wetting effectiveness factor using the modified Washburn equation.

Apparent shear viscosity of the polymeric solutions

The apparent shear viscosity of the aqueous solutions was measured using an R/S Plus Rheometer (Brookfield Engineering, Middleboro, MA, USA) with a water jacket assembly Lauda Eco (Lauda-Brinkmann LP, Delran, NJ, USA). A coaxial cylinder (CC40) was used to provide a controlled shear rate on the samples. A shear rate from 0 to 1000 1/s for 60 s was used for Kol–SDS and Soluplus–SDS solutions. A shear rate from 0 to 400 1/s for 60 s was used for HPC–SDS solution due to its high viscosity and limitation of our viscometer. The temperature of the jacket was kept constant at 25 ± 0.5 °C. The raw data were analyzed using the Rheo 3000 software (Brookfield Engineering, Middleboro, MA, USA) of the equipment to obtain the apparent shear viscosity as a function of the shear rate. The apparent shear viscosity at ~100 1/s was used as a representative low shear rate value.

Surface tension of the solutions

The surface tension of deionized water and the aqueous solutions was measured using Attension Sigma 700 (Biolin Scientific, Linthicum, MD, USA). The Attension software calculates surface tension from force measurements of interaction of a probe (Wilhelmy plate) at the boundary between air and a liquid, i.e., the deionized water or the solution.

Drug wettability with the solutions

Attension Sigma 700 set-up (Biolin Scientific, Linthicum, MD, USA) was used to study the penetration of water/aforementioned polymeric solutions into a packed powder bed of drug (griseofulvin, GF) particles inside a cylindrical column and determine the drug powder wettability, based on the Washburn method. The assembly consists of a sample holder in the form of a cylindrical metallic tube with small holes at the bottom as well as a hook at the top of the cover equipped with screw threads. About 0.8 g of GF

powder was packed uniformly into the tube before each measurement. A filter paper was placed at the perforated end of the sample holder to support the drug powder sample. A petri dish containing deionized water/polymeric solution was placed below the perforated end of the holder on the mechanical platform.

Upon contact of the sample holder with deionized water/polymeric solution, the liquid penetrated the drug powder bed, while Attension Sigma 700 recorded the mass of liquid penetrated the drug powder bed as a function of time. The contact angle for the deionized water/solution and drug can be determined using the modified Washburn equation, which provides a relationship between liquid penetration rate and contact angle θ, i.e., $M^2 = \left(C\rho^2\gamma\cos\theta/\eta\right)T$, where T, M, η, ρ, and γ are time after contact, mass of the liquid penetrated the drug powder bed, viscosity, density, and surface tension of the liquid, respectively. C is a characteristic parameter of the powder sample (GF powder in the current study), which could have been determined independently using a completely wetting liquid such as hexane, heptane, etc. Since the same drug powder (GF) was used as the powder sample and C depends only on powder packing, C remained invariant for different polymer solutions and deionized water studied. This allows us to calculate the ratio of $\cos\theta_{ss}/\cos\theta_w$ as a wetting effectiveness factor, in which θ_{ss} is the contact angle between GF and the polymer–SDS stabilizer solution and θ_w is the contact angle between GF and deionized water. The wettability enhancement upon the use of different stabilizers (polymers/surfactant) on the wetting of drug particles can be assessed by using this ratio, taking the wettability by water as a basis for comparison.

Experimental liquid penetration data (M^2 vs. T) for water and various stabilizer solutions are presented in Figure S1. The slope of the modified Washburn equation, i.e., $C\rho^2\gamma\cos\theta/\eta$, was obtained by fitting the linear region of the liquid penetration curve. Initial ~20 s was not considered due to transient behavior, and data points that deviated from the linear region, which may correspond to structural change in the bed, were excluded. The modified Washburn equation fitted the data almost perfectly ($R^2 \geq 0.990$). Using the slope for different stabilizer solutions and water, $\cos\theta_{ss}/\cos\theta_w$ was calculated. The viscosity, surface tension, and calculated wetting effectiveness factor are reported in Table 5 of the main text.

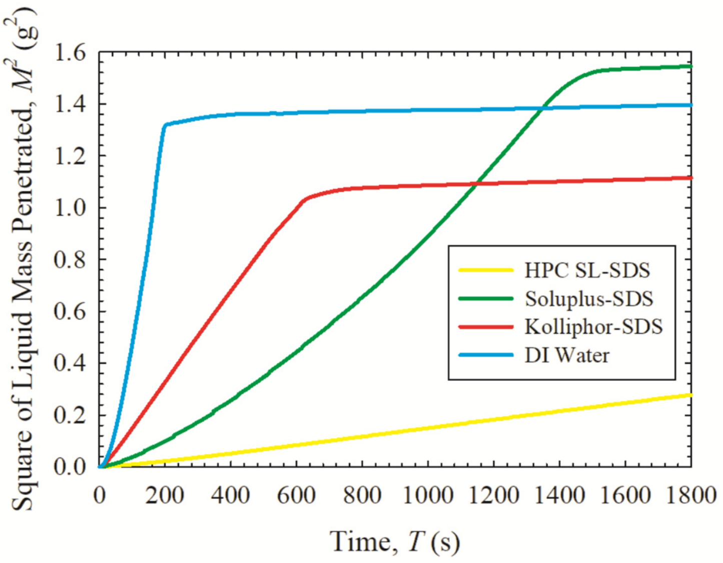

Figure S1. Temporal evolution of the liquid mass penetrated a packed bed of as-received GF particles by various aqueous stabilizer solutions and deionized (DI) water.

References

1. Hołownia, D.; Kwiatkowska, I.; Hupka, J. An investigation on wetting of porous materials. *Physicochem. Probl. Miner.* **2008**, *42*, 251–262.
2. Washburn, E.W. The dynamics of capillary flow. *Phys. Rev.* **1921**, *17*, 273.
